# Kidney injury molecule 1 in the early detection of acute kidney injury—a systematic review and meta-analysis

**DOI:** 10.3389/fmed.2025.1574945

**Published:** 2025-10-17

**Authors:** Yun Su, Xin Yang, Wei-Wei Cheng, Xue-Mei Shang, Hong-Lian Wang, Hong-Chun Shen

**Affiliations:** ^1^The Department of Nephrology, The Affiliated Traditional Chinese Medicine Hospital, Southwest Medical University, Luzhou, Sichuan, China; ^2^College of Integrated Chinese and Western Medicine, The Affiliated Traditional Chinese Medicine Hospital, Southwest Medical University, Luzhou, Sichuan, China; ^3^Research Center for Integrative Medicine, The Affiliated Traditional Chinese Medicine Hospital, Southwest Medical University, Luzhou, Sichuan, China

**Keywords:** kidney injury molecule 1, urinary KIM-1, serum KIM-1, acute kidney injury, meta-analysis

## Abstract

**Background:**

Kidney Injury Molecule 1 (KIM-1) is a biomarker of proximal tubular injury that can be used for the early detection of acute kidney injury (AKI). This study was designed to systematically review the relevant literature to assess the role of urinary KIM-1 (uKIM-1) and blood KIM-1 (bKIM-1) in diagnosing adult AKI.

**Methods:**

We searched PubMed, Embase, Cochrane, web of science for literature published until 7 August 2024, using the Quality Assessment Tool for Diagnostic Accuracy Studies (QUADAS-2). Sensitivity, specificity, and area under the curve (AUC) values from the included studies were combined using stata 18.

**Results:**

In total, 41 studies involving 1,790 patients were included. The estimated sensitivity of uKIM-1 for diagnosing adult AKI was 0.73 (95% CrI, 0.67–0.78), the specificity was 0.75 (95% CrI, 0.70–0.80), and the AUC was 0.81 (95% CrI 0.77–0.84); while the estimated sensitivity of bKIM-1 for diagnosing AKI was 0.72 (95% CrI 0.65–0.79), specificity was 0.79 (95% CrI, 0.70–0.86), and AUC was 0.81 (95% CrI 0.77–0.84).

**Conclusion:**

uKIM-1 and bKIM-1 show potential as biomarkers for predicting AKI in adult patients, demonstrating relatively high sensitivity and specificity. However, the current meta-analysis does not provide sufficient evidence to make definitive conclusions, and further studies and clinical trials are needed to determine the practical utility of uKIM-1 and bKIM-1 in clinical diagnosis.

## 1 Introduction

Recognized by almost all medical disciplines, acute kidney injury (AKI) is a common and serious condition. It is characterized by a dramatic decline in kidney function over a period of hours to days and is usually reversible (1). Many patients presenting with AKI have a mixed etiology, often consisting of coexisting sepsis and ischemia-reperfusion injury (2). It affects approximately 25% of hospitalized patients, particularly critically ill patients in intensive care units. It is estimated that AKI affects approximately 20–200 people per million population in the community, accounts for 7–18% of hospitalizations, and occurs in approximately 50% of patients in intensive care units (3, 4). And is associated with cardiovascular disease, end-stage renal disease (ESRD), hypertension, and death (5–7). AKI reportedly causes 2 million deaths worldwide each year, and AKI survivors are at increased risk of chronic kidney disease and ESRD, with significant economic, social, and personal burdens (8, 9). The urgent need for more accurate and effective diagnostic tools for AKI has been highlighted by these studies.

Several new AKI biomarkers have been found and characterized in the last few years. Some are thought to have potential to help diagnose AKI, including neutrophil gelatinase-associated lipid transport protein, interleukin-18, kidney injury molecule-1 (KIM-1), and tissue inhibitor of metalloproteinases 2 (10, 11). Among these various new biomarkers, many researchers have demonstrated that KIM-1 is a significant predictive marker for AKI detection.

KIM-1, as a type I transmembrane protein, was originally hypothesized to be an epithelial cell adhesion molecule, containing a novel immunoglobulin structural domain. In the normal state, this structural domain is typically absent, although its levels are observed to be heightened in proximal tubular parietal cells subsequent to renal tubular damage (12, 13). It has been proposed that uKIM-1 is a sensitive and specific marker of renal injury and can be employed as a prognostic predictor, particularly in the context of AKI in adult patients (14). Despite a substantial body of research, the clinical utility of KIM-1 for the early diagnosis of AKI remains to be established in larger, well-designed studies. Existing evidence is constrained by heterogeneity in patient populations and variability in the timing of biomarker measurement relative to the renal insult. Moreover, few studies have examined how different KIM-1 types in adult populations. Accordingly, this study aimed to evaluate the diagnostic performance of urinary and blood KIM-1 (uKIM-1 and bKIM-1) in adults with AKI.

## 2 Methods

We followed the Preferred Reporting Items for Systematic Reviews and Meta-Analyses (PRISMA) guidelines (15). under the registration identifier CRD42024580593.

### 2.1 Search strategy

Two independent researchers (YS and WWC) searched PubMed, Web of Science, EMBASE, and the Cochrane Library by searching literature searches up to August 2024. The search included keywords such as “Kidney Injury Molecule 1” or “KIM-1” plus “Acute Kidney Injury” or “Acute Kidney Failure.” In addition, a manual review of references to relevant studies was conducted. There were no language restrictions. In the event of disagreement, the problem was resolved through the involvement of a third researcher (XY). Detailed search formulas for each database are provided in Additional file 1.

### 2.2 Inclusion and exclusion criteria

Studies that met the following criteria were ultimately retrieved:(1) studies were conducted in patients with AKI, age >18 years; (2) articles with a prospective cohort design, case–control design, or cross-sectional design and explored the performance of uKIM-1 and bKIM-1 in the detection of AKI; and (3) studies that included or allowed for calculation of the estimated sensitivities of uKIM-1 and bKIM-1 in the diagnosis of AKI and the specificity studies.

Criteria for exclusion were: (1) no reported diagnostic accuracy measures for any biomarkers; (2) no reported AKI; and (3) conference abstract, PhD dissertation, review article, or other editorial; (4) pediatric studies.

### 2.3 Study selection

The retrieved literature was imported into Endnote X9 by two authors (YS and WWC). Duplicates were deleted, and the titles and abstracts of all retrieved articles were screened to exclude non-compliant articles. The final compliant literature was identified based on the inclusion criteria by reading the full text. In the event of disagreement, the problem was resolved through the involvement of a third researcher (XY).

### 2.4 Data extraction and quality assessment

The following information was extracted from each eligible study: first author, country of origin, year of publication, study design, sample size, patient characteristics (age, sex), and the number of patients who developed AKI. Additionally, data regarding KIM-1 were extracted, including the type of biomarker reported, time of measurement, sensitivity, specificity, true-positive, true-negative, false-positive, and false-negative values for each study.

The Quality Assessment Tool for Diagnostic Accuracy Studies (QUADAS-2) (16) This is a quality assessment tool that has been developed with the specific purpose of enabling the systematic evaluation of diagnostic accuracy studies in order to assess the potential for bias in the studies themselves (17), including 14 questions (each categorized as ‘yes’, ‘no’ or unclear).

The data extraction and quality assessment described above were conducted by two authors (YS and WWC) in an independent manner. In the event of a discrepancy, the matter was settled through the involvement of a third researcher (XY).

### 2.5 Statistical analyses

Statistical analyses were performed using STATA version 18.0 (Stata Corp, College Station, TX), notably with the “midas” commands (18). The bivariate mixed-effects model fits a 2-level model, with independent binomial distributions for the true positives and true negatives conditional on the sensitivity and specificity in each study, and a bivariate normal model for the logit transformations of sensitivity and specificity between studies (18). Based on this model, the pooled sensitivity, pooled specificity, positive likelihood ratio, negative likelihood ratio, and diagnostic odds ratio (DOR) with their 95% CrI were obtained. We also constructed hierarchical summary ROC curves to plot sensitivity versus specificity and calculated the AUC (19). The degree of heterogeneity, which indicates the variation of included studies, was assessed using the I^2^ statistic (20). I^2^ describes the percentage of total variation across studies that is attributable to heterogeneity rather than to chance. The value of I^2^ lies between 0 and 100%, a value of 0% indicates no observed heterogeneity, and values greater than 50% may be considered substantial heterogeneity (18). In addition, we conducted a subgroup analysis according to the detection time, sample size, and presence of chronic kidney disease. Publication bias was evaluated using Deek’s effective sample size funnel plot.

## 3 Result

### 3.1 Selection process

**Figure 1:**
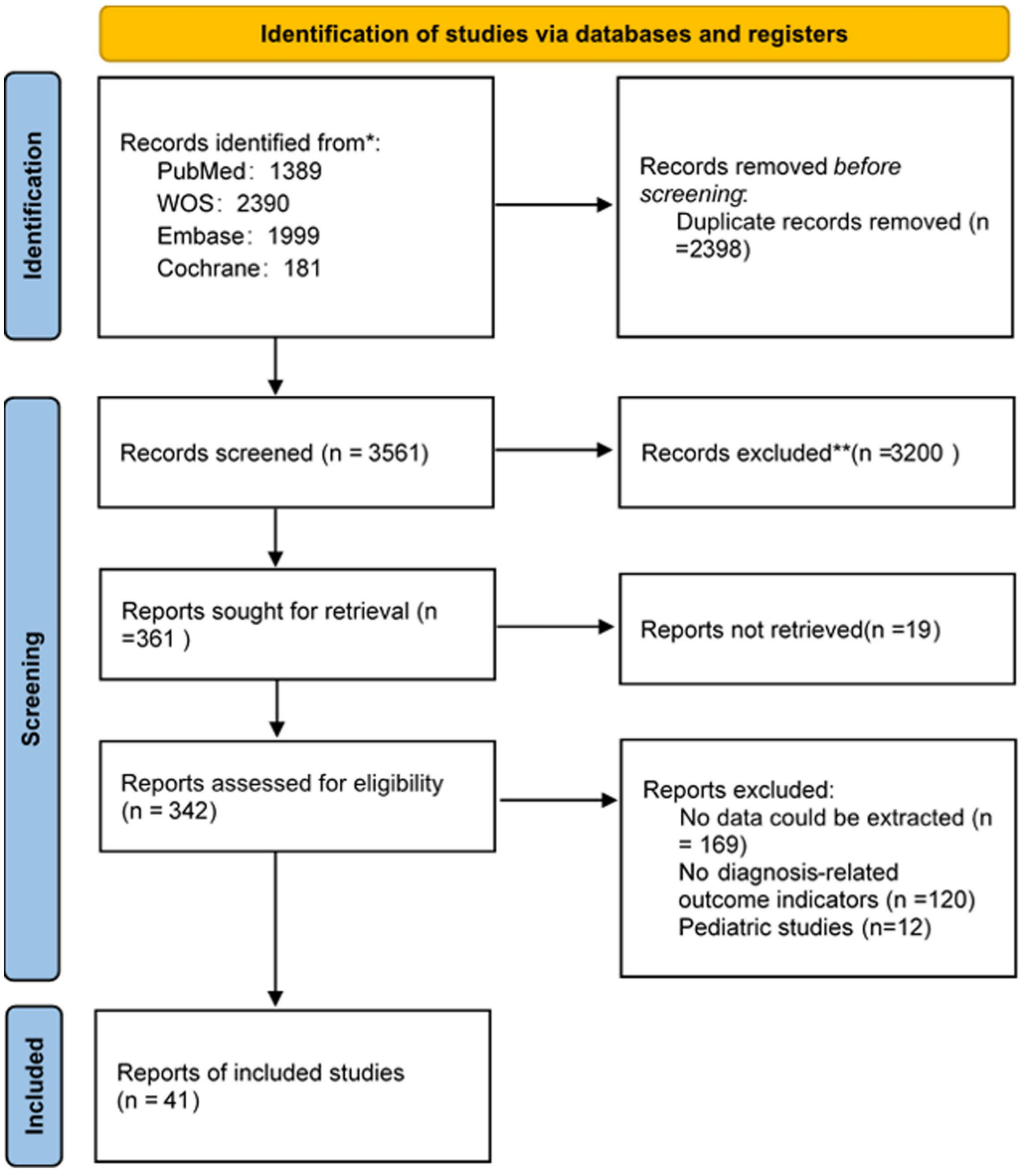
The study selection process.

The study selection process is shown in Figure 1. A total of 5,959 publications from different databases were retrieved upon initial search. Of those, 2,398 articles were excluded due to duplication. The remaining studies were screened by title and/or abstract; 3,219 of them were removed because they were reviews, animal research, or conference abstracts. Of the remaining 342 studies (corrected), 301 were excluded due to missing essential data (e.g., sensitivity, specificity, and diagnostic criteria used) and pediatric studies. In summary, 41 original studies (corrected) (21–62) were included in this meta-analysis.

### 3.2 Study characteristics

Table 1 summarizes 41 diagnostic studies. Across studies reporting age (*n* = 38), the study-level, unweighted mean age was 56.9 years, with a median of 58.6 years and an interquartile range (IQR) of 51.0–65.0 years (values outside 0–100 years were excluded as implausible). The median study sample size, estimated from the 2 × 2 data (TP + FP + FN + TN), was 149 patients (IQR 83–225; range 22–2,067).

**Table 1:** The characteristics of the individual studies.

Study	Country	Type	AKI diagnostic criteria	AKI patient age	Sensitivity	Specificity	TP	FP	FN	TN	Indicator
H.-M. PANG 2017 (36)	China	Case–control	NA	52.2 ± 19.5	1.02498	0.74967	11	19	0	57	uKIM-1-day0
					0.78787	0.90656	9	7	2	69	uKIM-1-day1
					0.85895	0.7455	9	19	2	57	uKIM-1-day2
					0.95714	0.83746	11	12	0	64	uKIM-1-day3
Jochen Metzger 2016 (33)	Germany	Case–control	AKIN criteria	65 ± 8	0.60	0.67	35	17	24	34	uKIM-1
Hongqi Ren 2015 (38)	China	Case–control	KDIGO criteria	33.4 ± 11.3	0.72672	0.80075	8	9	3	38	uKIM-1
David A. Jaques 2019 (28)	Switzerland	cohort study	AKIN criteria	60.7 ± 9.7	0.52962	0.55618	29	22	26	28	uKIM-1
Venkata S. Sabbisetti 2014 (39)	Britain	Cross-sectional study	KDIGO criteria	74 ± 2	0.82268	0.86005	23	9	5	55	Blood KIM-1
					0.90199	0.89518	25	7	3	57	uKIM-1
Chao-Wei Lee 2018 (30)	China	Cohort study	RIFLE criteria	> 25	0.692	0.778	15	2	7	9	Blood KIM-1
Chia-Hung Yang 2016 (54)	China	Cross-sectional study	KDIGO criteria	69 ± 2	0.80	0.44	39	30	10	24	uKIM-1
Hong-hua YE 2018 (55)	China	Case–control	NA	75.06 ± 8.31	0.52677	0.50031	8	16	8	16	uKIM-1-6 h
					0.52337	0.73081	8	9	8	23	uKIM-1-24 h
Rubina Naqvi 2023 (35)	Pakistan	Case–control	KDIGO criteria	28.53 ± 6.74	0.47601	0.46291	12	66	14	57	uKIM-1
Katherine Xu 2021 (52)	USA	Cohort study		63.7 ± 19.2	0.5214	0.47178	111	251	102	225	uKIM-1-AKI1-3
			AKIN criteria		0.62804	0.52907	53	285	31	320	uKIM-1-AKI2-3
					0.6199	0.48555	25	333	16	315	uKIM-1-AKI3
Zheng Shaoxiong 2022 (42)	China	Case–control	NA	57.27 ± 17.86	0.8636	0.8846	19	9	3	69	Blood KIM-1
Yuanyuan Xie 2016 (51)	China	Case–control	KDIGO criteria	53 ± 8.44	0.663	0.647	65	30	33	56	uKIM-1
Luis E. Morales-Buenrostro 2014 (34)	Me’xico	Case–control	AKIN criteria	54.5 ± 22.5	0.1	0.1	2	18	15	2	uKIM-1-day1
			KDIGO criteria		0.83	0.95	14	1	3	19	uKIM-1-day2
					0.722	0.633	18	53	7	92	uKIM-1
Manuel J. Vogel 2021 (49)	Germany	Cohort study		55.3 ± 17.2	0.75186	0.75395	10	16	3	51	uKIM-1
Nora A. Khreba 2019 (29)	Egypt	Cohort study	KDIGO criteria	47.40 ± 15.24	0.48	0.94	13	1	14	17	3 h Postoperative KIM-1
Frederik H 2013 (48)	Belgium	Cohort study	RIFLE criteria	64 ± 15	0.64657	0.60437	9	27	5	42	uKIM-1
Mohammed F Abosamak 2021 (58)	Egypt	Case–control	AKIN criteria	44.41 ± 10.67	0.42401	0.69099	31	36	42	79	uKIM-1
Maryam Saeedi Ghaheh 2021 (60)	Iran	Cross-sectional study	NA	50.04 ± 6.02	0.84	0.89	38	6	7	45	uKIM-1
C.-F. Zhang 2020 (56)	China	Cohort study	NA	66.88 ± 15.41	0.70151	0.46229	48	38	21	32	uKIM-1
					0.663	0.73514	46	19	23	51	Blood KIM-1
Wei Xue 2014 (53)	China	Cohort study	NA	53.00 ± 10.61	0.9	0.75	56	12	6	35	uKIM-1
Buket Kin Tekce 2015 (44)	Turkey	Cohort study	AKIN criteria	59.1 ± 6.7	0.875	0.933	7	1	1	13	uKIM-1-day1
Tiezhen Liu 2023 (21)	China	Cohort study	KDIGO criteria	39.96 ± 9.52	0.821	0.786	23	3	5	11	Blood KIM-1
Yuanyuan Pei 2022 (37)	China	Cohort study	KDIGO criteria	75 ± 59.84	0.68898	0.73141	41	27	19	75	Blood KIM-1
Mostafa Abdelsalam 2018 (22)	Egypt	Cohort study	KDIGO criteria	50.57 ± 13.33	0.9623	1	34	0	1	97	uKIM-1-Cut off < 1.685
					0.9623	0.9744	34	2	1	95	uKIM-1-Cut off < 1.730
					0.9811	0.9744	34	2	1	95	uKIM-1-Cut off < 1.750
WENHUA LI 2015 (32)	China	Case–control	NA	66.8 ± 9.9	0.737	0.857	14	18	5	108	uKIM-1-24 h
Michael A. Ferguson 2010 (25)	USA	Cross-sectional study	NA	60.5 ± 17.2	0.77	1	71	0	21	68	uKIM-1
Alexandra JM Zwiers 2015 (57)	Netherlands	Cohort study	RIFLE criteria	39.0 ± 0.65	0.51884	0.58198	18	27	17	38	uKIM-1 T0 0–6 h
					0.55614	0.68602	19	20	16	45	uKIM-1 T1 6–12 h
					0.68769	0.70323	24	19	11	46	uKIM-1 T2 12–24h
					0.54453	0.8321	19	11	16	54	uKIM-1 within 24 h
Dana Y. Fuhrman 2020 (26)	USA	Cohort study	KDIGO criteria	114 ± 42.25	0.83616	0.55375	5	4	1	6	uKIM-1 Prior to LTx
					0.83675	0.63798	5	4	1	6	uKIM-1 Within 6 h after LTx
					0.84509	0.81092	5	2	1	8	uKIM-1 24 h after LTx
Lei Lei 2018 (31)	China	Cohort study	KDIGO criteria	59.72 ± 10.16	0.634	0.816	43	15	25	67	uKIM-1
Dan Tan 2022 (43)	China	Cohort study	Kidigo clinical practice guidelines	52.83 ± 10.21	0.571	0.874	21	15	15	106	uKIM-1 4 h
					0.946	0.907	34	11	2	110	uKIM-1 12 h
					0.893	0.94	32	7	4	114	uKIM-1 24 h
					0.929	0.854	33	18	3	103	uKIM-1 48 h
Phurailatpam Uma Devi 2022 (59)	India	Cohort study	KDIGO criteria	62.33 ± 5.32	1	0.636	6	16	0	28	Blood KIM-1
Said M. Elmedany 2017 (24)	Egypt	Cohort study	AKINcriteria	60.5 ± 6.6	0.654	0.592	7	14	4	20	uKIM-1 after induction
					0.545	0.588	6	14	5	20	uKIM-1 2 h after CPB
					0.636	0.529	7	16	4	18	uKIM-1 6 h after CPB
					0.818	0.765	9	8	2	26	uKIM-1 12 h after CPB
					0.636	0.706	7	10	4	24	uKIM-1 24 h after CPB
Maciej T. Wybraniec MD 2017 (50)	Poland	Cohort study	AKIN criteria	65 ± 3	0.889	0.75	8	22	1	65	uKIM-1
					0.778	0.824	7	15	2	71	uKIM-1 6 h
Mona Schaalan 2017 (40)	Egypt	Case–control	KDIGO criteria	45–51	0.64676	0.7532	26	10	14	30	Blood KIM-1
Amr Mohamed Shaker 2023 (41)	Egypt	Cohort study	NA	55.55 ± 10.73	0.564	1	23	0	17	40	Blood KIM-1
Won K. Han 2009 (27)	USA	Cohort study	AKIN criteria	68.31 ± 2.30	0.5143	0.7778	19	12	17	42	uKIM-1 at Post immediately > 1.2 ng/mg Ucr
					0.4286	0.8889	15	6	21	48	uKIM-1 at Post immediately > 1.8 ng/mg Ucr
					0.3571	0.9020	13	5	23	49	uKIM-1 at 3 h Post-OP > 1.2 ng/mg Ucr
					0.3214	0.9608	12	2	24	52	uKIM-1 at 3 h Post-OP >1.8 ng/mg Ucr
Isidro Torregrosa 2014 (45)	Spain	Case–control	RIFLE	72 ± 10	0.69833	0.56689	10	15	5	19	uKIM-1
					0.54818	0.77864	11	27	9	97	uKIM-1
Yuexing Tu 2014 (46)	China	Case–control	AKIN criteria	58 ± 9	0.48	0.18	24	83	25	18	uKIM-1 0 h
					0.50	0.40	25	61	25	40	uKIM-1 1 h
					0.46	0.5	23	51	26	51	uKIM-1 3 h
					0.94	0.61	46	39	3	62	uKIM-1 6 h
					0.91	0.78	45	22	4	79	uKIM-1 24 h
					0.89	0.62	44	38	5	63	uKIM-1 48 h
Xin-Ling Liang 2010 (61)	China	Case–control	RIFLE criteria	30 ± 5.5	0.933	0.739	28	24	2	68	uKIM-1 at 6 h > 1.5
					0.767	0.783	23	20	7	72	uKIM-1 at 6 h > 2.0
					0.9	0.728	27	25	3	67	uKIM-1 at 12 h > 1.5
					0.9	0.783	27	20	3	72	uKIM-1 at 12 h > 2.0
Noa Berlin 2024 (23)	USA	Cohort study	KDIGO	70 ± 5	0.58107	0.49059	31	50	23	49	Blood KIM-1
Mahryar Mehrkesh 2022 (62)	Iran	Case–control	RIFLE criteria	NA	0.56913	0.7954	13	5	9	18	Blood KIM-1 8 h
					0.76245	0.65395	17	8	5	15	Blood KIM-1 4 d

KIM-1, kidney Injury Molecule 1; uKIM-1, urinary KIM-1; AKI, acute kidney injury; KDIGO criteria, Improving Global Outcomes; RIFLE criteria.

Regarding design, cohort studies comprised 53.7% (22/41), case–control studies 36.6% (15/41), and cross-sectional studies 9.8% (4/41). AKI diagnostic criteria were most commonly KDIGO (39.0%, 16/41), followed by AKIN (22.0%, 9/41) and RIFLE (14.6%, 6/41); criteria were unreported in 24.4% (10/41). Studies were conducted across 15 countries; the largest contributions came from China (16/41, 39.0%), Egypt (6/41, 14.6%), and the USA (5/41, 12.2%), with additional single- or two-study contributions from Germany, Iran, the Netherlands, Turkey, Switzerland, Belgium, Spain, and others.

Most diagnostic evaluations focused on urinary KIM-1 (uKIM-1) (66 data rows across various time windows), whereas blood/serum KIM-1 (bKIM-1) accounted for 12 evaluations, reflecting a predominance of urinary measurements and multiple predefined sampling time points. Full per-study details, including country, design, AKI criteria, age reporting, and 2 × 2 diagnostic counts, are provided in Table 1.

### 3.3 Quality assessment

**Figure 2:**
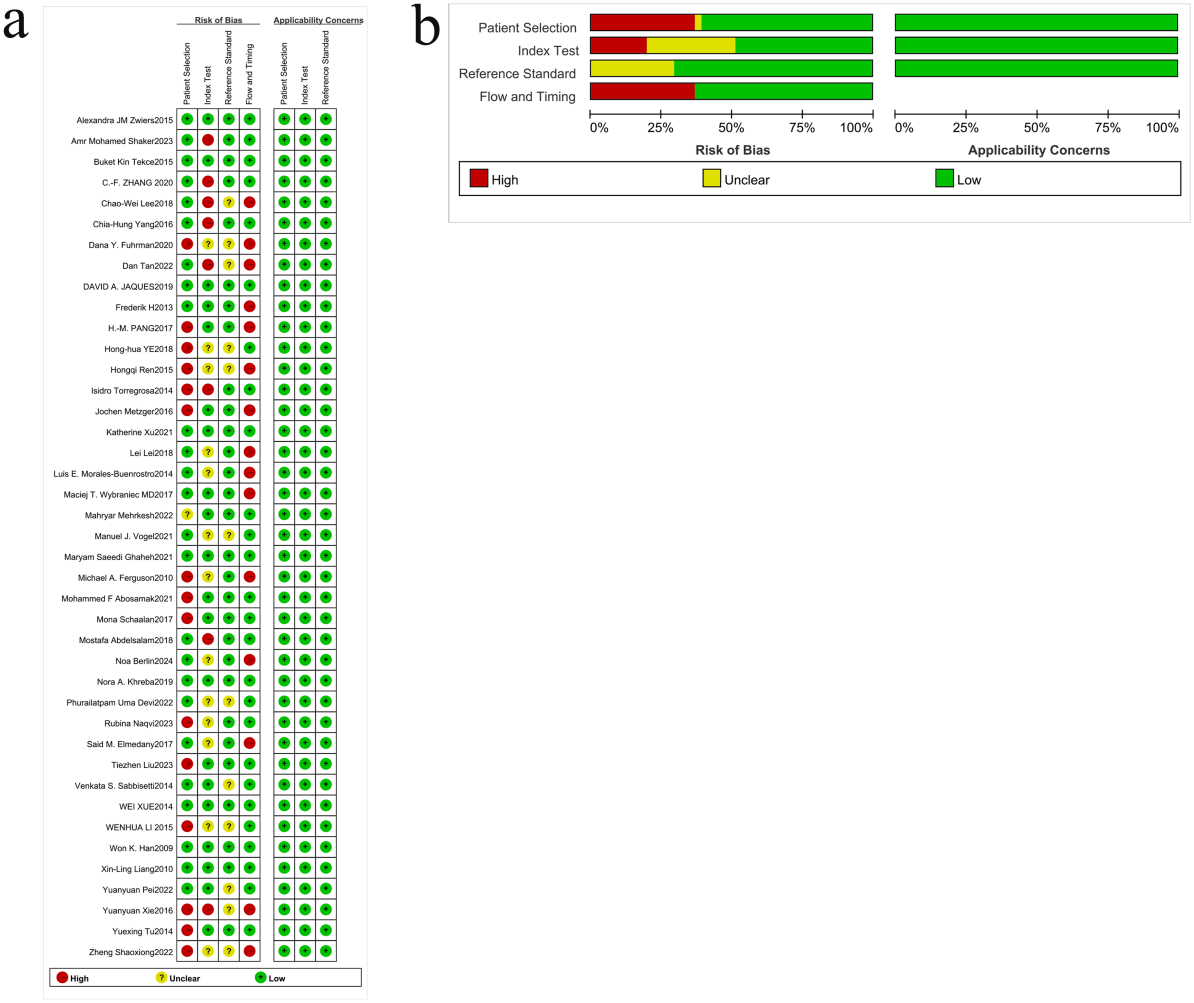
Quality assessment of the included studies. **(a)** Methodological quality graph. **(b)** Methodological quality summary.

We assessed risk of bias using QUADAS-2 across four domains and evaluated concerns regarding applicability in 41 diagnostic accuracy studies (Figure 2). Domain-level judgments were as follows (*n*, %):

*Patient selection*: low 25 (61.0%), high 14 (34.1%), unclear 2 (4.9%).
*Index test*: low 20 (48.8%), high 8 (19.5%), unclear 13 (31.7%).
*Reference standard*: low 23 (56.1%), high 0 (0%), unclear 18 (43.9%).
*Flow and timing*: low 20 (48.8%), high 9 (22.0%), unclear 12 (29.3%).
*Concerns regarding applicability (overall)*: low 25 (61.0%), high 9 (22.0%), unclear 7 (17.1%).

Common sources of bias included non-consecutive or non-random case inclusion and the use of case–control designs (patient selection), non-blinded interpretation of the index test and *post hoc* threshold selection (index test), insufficient reporting of blinding or the discriminative capacity of the reference standard (reference standard), and variable/ differential verification, heterogeneous intervals between index and reference tests, or exclusion of participants from the analysis (flow and timing). Applicability concerns mainly reflected mismatches between study populations or testing procedures and routine clinical pathways.

Overall, while over half of the studies were judged at low risk in several domains, the substantial proportions of high or unclear risk—particularly for the index test, reference standard reporting, and flow/timing—underscore the need for future studies with consecutive sampling, prespecified thresholds, blinded interpretation, uniform verification, and complete case inclusion to strengthen internal validity and clinical generalizability.

### 3.4 Data analysis

#### 3.4.1 uKIM-1

The diagnostic effect of uKIM-1 in patients with AKI was investigated in 32 studies.

**Figure 3:**
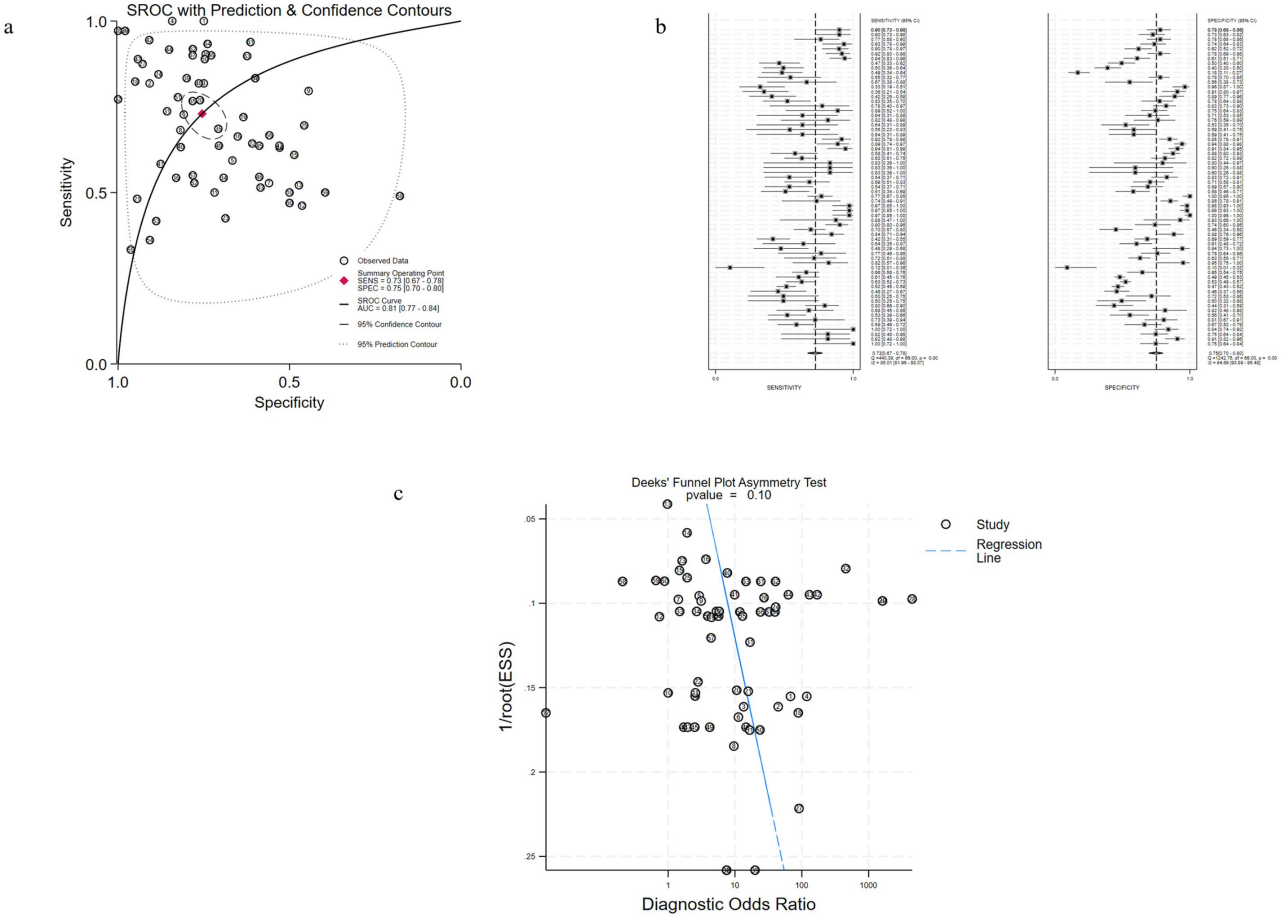
Performance of uKIM-1 for AKI diagnosis in Studies Included in the meta-analysis. **(a)** SROC plots of uKIM-1 to predict adult AKI. **(b)** Forest plots of the pooled sensitivity and specificity of uKIM-1 to predict adult AKI. **(c)** Funnel plot for the evaluation of potential publication bias in diagnosis of uKIM-1 for adult AKI.

The diagnostic effect of uKIM-1 in adult patients with AKI was investigated in 32 of these studies (Figures 3a–c). The results showed that the estimated diagnostic sensitivity of uKIM-1 was 0.73 (95% CrI, 0.67–0.78), the specificity was 0.75 (95% CrI, 0.70–0.80) and the DOR was 8 (95% CrI, 5–13), as shown in Figure 3 and Table 2. The I^2^ indices were 85.01% (81.96–88.07%) and 94.69% (93.89–95.48%), respectively. The sROC results showed an AUC of 0.81 (95% CrI, 0.77–0.84) for uKIM-1, with substantial heterogeneity in both sensitivity and specificity between studies. The funnel plot showed no significant publication bias (*p* = 0.1).

**Table 2:** Pair-wise comparisons between modalities for sensitivity, specificity, PLR, NLR, and AUC.

Category	Sensitivity	*P*	Specificity	*P*	PLR	*P*	NLR	*P*	AUC	*P*
uKIM-1	0.73 [0.67, 0.78]	NA	0.75 [0.70, 0.80]	NA	3 [2.3, 3.8]	NA	0.36 [0.28, 0.45]	NA	0.81 [0.77–0.84]	NA
bKIM-1	0.72 [0.65, 0.79]	NA	0.79 [0.70, 0.86]	NA	3.4 [2.3, 5.2]	NA	0.35 [0.26, 0.47]	NA	0.81 [0.77–0.84]	NA

The results indicated that the predictive value of uKIM-1 is regarded as the potential biomarker in adult AKI patients.

#### 3.4.2 bKIM-1

**Figure 4:**
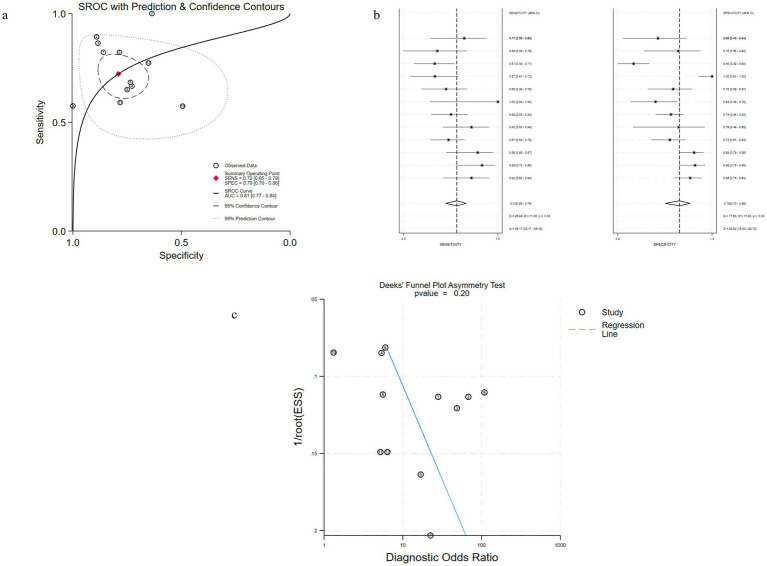
Performance of bKIM-1 for AKI diagnosis in Studies Included in the meta-analysis. **(a)** Hierarchical summary receiver operating characteristic (SROC) plots of sKIM-1 to predict adult AKI. **(b)** Forest plots of the pooled sensitivity and specificity of bKIM-1 to predict adult AKI. **(c)** Funnel plot for the evaluation of potential publication bias in diagnosis of uKIM-1 for adult AKI.

Among them, 10 studies involved patients with adult AKI diagnosed by bKIM-1 (Figures 4a–c), and the results showed that the estimated sensitivity of bKIM-1 diagnosis was 0.72 (95% CrI 0.65–0.79) and the specificity was 0.79 (95% CrI, 0.70–0.86). The combined diagnostic ratio (DOR) was 10 (95% CrI, 5–19) and the AUC of bKIM-1 for the diagnosis of AKI was 0.81 (95% CrI 0.70–0.86). The funnel plot showed no significant publication bias (*p* = 0.20).

## 4 Discussion

Early diagnosis of AKI plays an essential role in its treatment and prognosis. Currently, AKI is commonly diagnosed by elevated serum creatinine or decreased urine output. However, Serum creatinine is less useful in AKI since patients are not in a stable condition, causing it to significantly lag behind actual kidney damage (63). It is urgent to find a more effective approach to measuring the diagnosis of AKI. Novel biomarkers have been suggested to have the potential to facilitate early diagnosis of AKI, among which is KIM-1. Previous literature reviews have shown that uKIM-1 levels are linked to the extent of kidney tissue damage and may serve as a dependable predictor of negative renal outcomes in acute tubular injury (64). Additionally, KIM-1 is a sensitive marker for AKI (65). All published studies assessing the diagnostic value of KIM-1 were included in this meta-analysis, and a total of 41 eligible studies were identified and data extracted. In this study, the performance of uKIM-1 and bKIM-1 in predicting AKI was evaluated using sensitivity, specificity, and AUC metrics. The results showed that both uKIM-1 and bKIM-1 were the one of promising predictors. The AUC values of all studies were greater than 0.80, showing a relatively good diagnostic value.

In specific studies, the findings by Geng et al. (14) showed that the AUC of uKIM-1 for diagnosing AKI in adults was 0.62 (95% CrI: 0.41–0.76); the findings by Shao et al. (66) indicated that the AUC of uKIM-1 for diagnosing AKI was 0.86 (95% CrI: 0.83–0.89); and the result from Fazel et al. (67) suggested that the AUC of uKIM-1 for predicting AKI in children was 0.69 (95% CrI: 0.62–0.77). However, in clinical diagnostic studies, bKIM-1 has only been found to have modest results (68). Cai (64) found that uKIM-1 correlates with renal tKIM-1 expression and tubular histologic injury in AKI; in ATI, it may aid prognostication, and combining uKIM-1 with bKIM-1 modestly improves discrimination for severe ATI. The results of our study suggest that uKIM-1 has a better diagnostic value in adults. According to the data of the experimental studies on animals, KIM-1 expression in the epithelial cells of the renal proximal tubules as well as its concentration in urine and blood plasma correlate with the severity of the pathological process in the kidneys (69). Elevation of KIM-1 level in urine (uKIM-1) is a more sensitive indicator of AKI than the reduction of creatinine clearance or albuminuria (70). In addition, it has been suggested that KIM-1 may exert a significant role in renal recovery and tubular regeneration after AKI (71, 72).

The benefits of this study include the first systematic assessment of the diagnostic value of different KIM-1 types in patients with AKI, and a comprehensive analysis of both urine and blood forms of KIM-1 to further clarify the similarities and differences in their diagnostic efficacy. However, there are some limitations to this meta-analysis. Limitations include the limited number of bKIM-1 studies in underage populations, the heterogeneity of sampling windows, and the assay/platform variability and incomplete reporting of normalization (uKIM-1/Cr), which collectively temper the generalizability of pooled estimates. These issues highlight the need for multicenter, platform-harmonized studies with standardized sampling windows and context-specific thresholds across ICU, perioperative, oncology, and pediatric settings.

## 5 Conclusion

In summary, our study found that uKIM-1 and bKIM-1 are promising predictors of AKI, especially in adult patients, with relatively high sensitivity and specificity. However, further studies and clinical trials are still needed to confirm whether and how KIM-1 is widely used for clinical diagnosis. In the future, we expect KIM-1 or other renal biomarkers to be fully applied in AKI, from clinical detection to treatment and even prevention. Nevertheless, these findings should be interpreted with caution due to heterogeneity in study designs, AKI definitions, sample timing, and assay variability. Further large-scale, high-quality prospective studies are required to validate the clinical utility of KIM-1.

## Data Availability

The original contributions presented in the study are included in the article/supplementary material, further inquiries can be directed to the corresponding author.
